# Plane Segmentation in Sensor-Acquired 3D Point Clouds Using Supervoxel-Based Geometric Constraints

**DOI:** 10.3390/s26061816

**Published:** 2026-03-13

**Authors:** Xiaohua Ran, Xu Ning, Qing An, Xijiang Chen

**Affiliations:** 1 East China Branch of Gezhouba Group, Shanghai 201100, China; ranxiaohua2424@163.com; 2 School of Safety Science and Emergency Management, Wuhan University of Technology, Wuhan 430070, China; 3 School of Artificial Intelligence, Wuchang University of Technology, Wuhan 430223, China; 120160450@wut.edu.cn (Q.A.); cxj_0421@163.com (X.C.)

**Keywords:** plane segmentation, projection line fitting, supervoxel adjacency relationship

## Abstract

Plane segmentation of real-world 3D point clouds captured by LiDAR or depth sensors remains challenging due to data sparsity, noise, and complex geometric configurations such as stepwise and intersecting non-coplanar structures. To address these issues inherent in sensor-acquired data, this paper proposes a geometry-aware plane segmentation method that leverages supervoxel boundary adjacency, normal coherence, and projection-line fitting constraints. Supervoxels were generated using the toward better boundary preserved supervoxel segmentation (TBBS) algorithm, and their natural adjacency relationships were constructed based on boundary points. Subsequently, the supervoxels were initially clustered according to their normal information. Finally, the projected point clouds of adjacent supervoxel were fitted with straight lines, and the fitting errors were calculated to optimize the clustering results. Experimental results demonstrate that this method performs excellently in handling stepwise non-coplanar structures, effectively segmenting planar regions with significant geometric features. It shows particular advantages in cases involving stepwise non-coplanar and intersecting planes. On benchmark datasets, the method achieves precision and recall rates of (97.7%, 94.4%, 91.2%, 80.4%, 92.3%) and (98.9%, 95.7%, 93.7%, 84.8%, 96.0%), respectively, highlighting its effectiveness and robustness for practical 3D sensing applications.

## 1. Introduction

Point clouds, as an important form of spatial data, are widely used in various fields such as autonomous driving [1], architectural modeling [2], robot navigation [3] and geographic information systems [4]. In these application scenarios, point cloud plane segmentation [5] is a fundamental and crucial task, aiming to extract planar structures with significant geometric features from sparse and irregular point cloud data. Plane segmentation contributes to the geometric understanding and modeling of scenes [6] and provides reliable prior information for object detection [7], point cloud registration [8], and three-dimensional (3D) reconstruction [9]. However, designing an efficient and robust plane segmentation algorithm has become an important research challenge due to the presence of noise, outliers, uneven distribution, and occlusion in point cloud data. With the rapid development of deep learning technology [10], many deep learning-based methods have made remarkable progress in point cloud segmentation [11]. However, in some specific application scenarios, traditional non-learning-based methods [12,13] still demonstrate unique advantages. Compared with deep learning methods, traditional approaches usually rely on the geometric features and spatial structure of point clouds for segmentation. They neither require a substantial amount of labeled data nor suffer from inefficiencies when operating in environments with limited computing resources. Traditional point cloud segmentation methods can be roughly divided into three categories: geometry-based methods, clustering-based methods, and rule-based methods.

The aforementioned challenges are particularly pronounced in real-world applications driven by 3D sensing technologies, such as LiDAR and depth cameras. Data acquired from these sensors is inherently noisy, sparse, and often contains complex non-coplanar structures like steps and intersections, which pose significant difficulties for robust scene understanding. While supervoxel-based methods like VCCS [14] and region-growing techniques such as LCCP provide a strong foundation for segmentation by grouping local points, they typically rely on a single criterion—such as normal vector differences or convexity—to guide merging. This reliance on local cues makes them susceptible to noise and ambiguous geometry at object boundaries, particularly in complex non-coplanar scenarios. For instance, a normal-based constraint alone may fail to distinguish between two distinct steps of a staircase, as their adjacent surfaces are nearly parallel, leading to under-segmentation. To address this gap, this paper proposes a robust three-dimensional plane segmentation method tailored to the complexities inherent in sensor-acquired point clouds. It first constructs natural adjacency relationships for the generated super voxels by using their boundary points. Then, a novel dual-constraint clustering strategy is employed: initial grouping based on normal coherence is subsequently refined by evaluating the overall fitting error of projected point clouds from adjacent supervoxels. This hierarchical approach ensures that only geometrically consistent regions are merged. The main contributions of this method are as follows:

(1) Using normal vector info and spatial transformation features of supervoxels, this method calculates boundary points and builds adjacency relations, enhancing segmentation accuracy and robustness.
(2) This method jointly constrains super voxel clustering via normal vector spatial relations and overall fitting errors after projection-fitting adjacent supervoxels, performing well in complex scenarios and on noisy data, especially for certain planes.

## 2. Related Work

Geometry-based plane segmentation methods leverage geometric features of sensor-acquired point clouds—such as normals and curvatures—for accurate segmentation. Traditional approaches such as RANSAC [15] and its variants (PROSAC [16], eRANSAC [17]) improve efficiency through optimized sampling and consistency evaluation, particularly in noisy conditions. Other methods directly utilize geometric relationships. For instance, reference [18] which combines region-growing with geometric features for efficient plane extraction in complex scenes (e.g., buildings, roads). The Hough transform and its variants [19] are also widely applied for shape detection, demonstrating resilience to noise and occlusion across domains. While geometry-based plane segmentation algorithms demonstrate remarkable robustness and simplicity in specific contexts, they encounter several limitations as datasets expand and application scenarios grow more complex. In particular, these methods often involve high computational complexity, exhibit significant sensitivity to parameter space discretization, and face substantial challenges in adapting to intricate environments.

Clustering-based methods accomplish plane or object segmentation through the process of grouping point clouds into clusters that possess similar characteristics. The DBSCAN algorithm [20] is a density-based clustering approach proficient in detecting clusters of arbitrary shapes and effectively handling noise. A Smiti et al. [21] proposed the DBSCAN-GM method, which combines the advantages of Gaussian mean and DBSCAN to address the limitations of DBSCAN. DBSCAN-GM can automatically generate appropriate parameters, handle noisy data, and produce high-quality clustering results. Chen et al. [22] proposed the KNN-BLOCK DBSCAN algorithm, which employs k-nearest neighbors techniques to identify core points and blocks. By processing data through dynamically ranged blocks, it significantly enhances the efficiency of DBSCAN. Consequently, this algorithm demonstrates high efficiency and accuracy when dealing with large-scale datasets.

Region growing is a clustering algorithm based on the similarity of points. In the work presented in [23], the authors introduced a normal-based region growing approach with automatic parameter selection via heuristic optimization, enabling efficient processing of large-scale point clouds and hierarchical multi-level segmentation to handle diverse object geometries. Huang et al. [24] proposed a multi-scale region growing method using multi-resolution supervoxels to enhance plane segmentation in noisy urban scenes, adapting to varying planar sizes and noise levels through multi-scale strategies. While region growing is conceptually simple and effective, its performance heavily relies on proper seed point selection and parameter tuning, limiting robustness when facing uneven point distributions or significant noise.

Supervoxels have become a powerful tool for sensor-acquired point cloud processing due to their efficiency and adaptability. For instance, in [14], an innovative voxel connectivity (VCCS) model was introduced, which utilized color similarity and geometric features for segmentation. However, its performance depends on parameter tuning, seed point initialization, and fixed voxel resolution, often producing unnatural rectangular voxel blocks. To address these limitations, reference [25] proposed the toward better boundary preserved supervoxel segmentation (TBBS) algorithm, which eliminates seed points and transforms segmentation into a subset selection problem by finely modeling local features to better preserve object boundaries. Building on VCCS, [26] enhanced segmentation accuracy by combining geometric and color features, while [27] adopted multi-scale analysis to optimize supervoxel clustering and avoid over-segmentation, guided by the quasi-a-contrario theory.

Rule-based point cloud segmentation methods employ manually designed rules or heuristic strategies by analyzing geometric features, spatial structures, and point attributes (e.g., normals, curvature, density). Ali Khaloo et al. [28] developed a segmentation framework using normal and distance consistency rules combined with region growing, achieving robust segmentation results. To address the issues of under-segmentation and boundary point loss inherent in traditional Optimal Vector Field (OVF) methods, Li et al. [29] proposed a Detail-Preserving OVF (DP-OVF) framework that integrates quasi-a-contrario-based fine planar primitive extraction with a novel point-based Laplace operator and a magnitude-driven region growing strategy; while this approach significantly improves segmentation precision and recall by preserving sharp structural edges and ensuring boundary completeness without the resolution constraints of voxelization, its computational efficiency remains limited for large-scale datasets due to the iterative optimization process, and performance can be sensitive to local point density variations when determining neighborhood parameters. Li et al. [30] proposed an efficient approach integrating RANSAC with Normal Distribution Transformation (NDT) Cells, which segment the point cloud based on local geometric features to capture points with similar normals, thereby improving planar region identification accuracy. These methods are easily implemented and allow precise algorithm control, though their performance may degrade in complex scenes. Huang et al. [31] proposed a unified Random Sample Consensus (RANSAC) framework that integrates point and line features through an adaptive mixed sampling strategy and a line-constrained boundary optimization mechanism; while this approach significantly enhances segmentation accuracy at structural edges and reduces computational runtime in urban scenarios by leveraging linear guidance, its performance remains inherently sensitive to the predefined inlier distance threshold and may yield suboptimal results in natural scenes lacking distinct linear features.

While supervoxel-based methods have proven effective, the criteria used for subsequent merging or region-growing are critical for final segmentation accuracy. The VCCS algorithm [14] generates supervoxels based on geometric and color similarity but often relies on simple fixed-depth adjacency for clustering, which may not accurately reflect complex object boundaries. Region-growing methods, including the implicit principles behind Locally Convex Connected Patches (LCCP), typically merge adjacent primitives based on local convexity or normal angles [23,24]. Although effective for smoothly varying surfaces, these local constraints can be ambiguous in the presence of noise or at the junctions of complex structures like stepwise or intersecting planes. For example, a pure normal-based constraint might erroneously merge two distinct but parallel planar surfaces belonging to different steps of a staircase, as the normal vectors at their boundary are nearly identical. Similarly, local curvature estimates can be unstable near step edges. In contrast, our proposed method introduces a novel global geometric verification step. After an initial clustering based on normal coherence, we project the point clouds of adjacent supervoxel clusters onto a shared plane and assess their linear fit. This global error metric provides a more robust test for true coplanarity, effectively distinguishing between adjacent planes that are geometrically separate despite similar local features, thereby overcoming a key limitation of previous approaches.

## 3. Proposed Method

**Figure 1:**
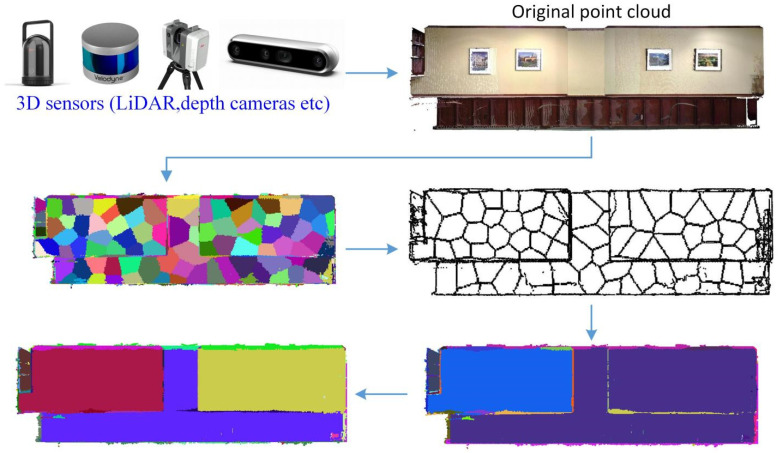
Flow chart of the proposed plane segmentation algorithm in sensor-acquired 3D Point Clouds.

To address the challenges posed by real-world 3D point clouds acquired from LiDAR or depth sensors—such as noise, sparsity, and complex geometric transitions—this study presents a novel three-dimensional plane segmentation framework comprising three key stages. First, Supervoxel Generation: Supervoxels with well-defined boundaries are extracted using the TBBS method, ensuring precise spatial structure preservation. Second, Clustering via Adjacency-Aware Supervoxel Relationships: Adjacency relations between supervoxels are established based on boundary points [32], followed by weighted clustering to respect intrinsic data topology. Third, Optimization through Plane Projection Fitting: Each supervoxel’s principal plane is determined via its covariance matrix’s largest eigenvalue. Projections onto this plane are fitted using Total Least Squares (TLS), and fitting errors refine the clustering, improving segmentation robustness—particularly for stepwise non-coplanar structures commonly encountered in real sensor acquisitions. The chart of the proposed plane segmentation algorithm in sensor-acquired 3D Point Clouds is shown in Figure 1.

### 3.1. Adjacency Connectivity

Supervoxel segmentation is a density-based approach that partitions point cloud data into spatially coherent regions sharing similar geometric properties. In this work, the TBBS algorithm is employed to generate supervoxels that effectively capture local structural information in raw point clouds—particularly those acquired from 3D sensors such as LiDAR or depth cameras, which often exhibit non-uniform sampling and boundary ambiguity. The TBBS method requires no seed-point initialization and is largely parameter-free, making it well-suited for processing real-world sensor data with varying densities.

Supervoxel adjacency relationships define the spatial proximity between supervoxels. The proposed boundary-based adjacency preservation method maintains the natural structural relationships between supervoxels, consistent with human visual perception. This work determines adjacency by identifying point pairs with minimal spatial distance between boundary points of adjacent supervoxel blocks. A systematic traversal of all boundary points yields a complete set of adjacency pairs, each characterized by shared boundary relationships. This process generates precise and reliable adjacency relationships that are essential for accurate data analysis and interpretation. The corresponding algorithm is presented in Algorithm 1.
**Algorithm 1**. Adjacency Generation**Input**: supervoxel SV; boundary points SB; adjacency radius R; candidate neighbors CN; **Output**: supervoxel adjacency, AD;**1. Extract boundary points:** **for all** $S_{i}\in SV$ **do**
   **for all** $p_{i}\;\in S_{i}$
**do**
    
**if** is_boundary **then**
      
$SB.add\left(p_{i}\right)$
**2. Find closest boundary point pairs:** **for all** $SB\left(S_{i}\right)\;\in \;SB\left(SV\right)$ **do**
   
**for all** $p_{i}\;\in SB\left(S_{i}\right)$ **do**

      
**for all** $p_{j}\;\in \;SB\left(CN\right)\;$
**do**

       
$\min\left\{distance\left(p_{i},p_{j}\right)\right\}$
       
$AD.isert\left(label\left(S_{i},S_{j}\right)\right)$**3. Filter adjacency pairs:** Remove all duplicate entries from **AD** **return AD**

For each supervoxel block $S_{i}\left(i=1,2,\ldots ,m\right)$, the normal vector $\nu_{1}\;\left(a,b,c\right)$ of the supervoxel block is determined through plane fitting, and the vector between the target point and the neighboring points in the supervoxel block is constructed, as shown in Equation (1):

(1) $$V_{ij}=O_{ij}-O_{i}$$

In the equation, $O_{i}$ is the target point, and $O_{ij}$ is the neighboring point of the target point. The magnitude of each vector is calculated based on Equation (1), and the unit vector is obtained:

(2) $${\hat{V}}_{ij}=\frac{V_{ij}}{\left|V_{ij}\right|}$$

And the vector with the longest magnitude, $V_{X}$ is taken as the coordinate’s north direction (vertical axis):

(3) $${\hat{V}}_{X}=\frac{V_{X}}{\left|V_{X}\right|}$$

According to the orthogonality principle, the east direction (horizontal axis) can be obtained, as shown in Equation (4):

(4) $$V_{Y}=\frac{{\hat{V}}_{X}\times \nu_{1}}{\left|{\hat{V}}_{X}\times \nu_{1}\right|}$$

Using the previously determined vertical axis direction ${\hat{V}}_{X}$ and horizontal axis direction $V_{Y}$, project all the vectors $V_{ij}$ of each target point in the supervoxel block onto the vertical and horizontal axes:

(5) $$\left\{\begin{array}{c} L_{ij}^{X}={\hat{V}}_{X}\cdot V_{ij}\\ L_{ij}^{Y}=V_{Y}\cdot V_{ij} \end{array}\right.$$

The azimuth angles of the neighboring vectors can be obtained from Equation (5), as shown in Equation (6):

(6) $$\alpha_{ij}=arctan\left(\frac{L_{ij}^{X}}{L_{ij}^{Y}}\right)$$

The azimuth angles obtained from Equation (6) may be negative. Therefore, the final azimuth angles are obtained using Equation (7):

(7) $$\left\{\begin{array}{c} {\alpha^{\prime }}_{ij}=\alpha_{ij},\alpha_{ij}\geq 0\;\\ {\alpha^{\prime }}_{ij}=\alpha_{ij}+360{}^{\circ},\alpha_{ij}<0\; \end{array}\right.$$

The azimuth angles ${\alpha \prime }_{ij}$ are sorted, and the differences between adjacent angles are calculated:

(8) $${∆}_{{\alpha^{\prime }}_{ij}}={\alpha^{\prime }}_{ij}-{\alpha^{\prime }}_{i(j-1)}$$

The boundary points of each supervoxel block are determined based on the maximum value of ${∆}_{{\alpha^{\prime }}_{ij}}$, as shown in Equation (9):

(9) $$\left\{\begin{array}{c} \mathrm{Boundary}\;\mathrm{point},\;\mathrm{if}\;\underset{j=1,2,\ldots ,k-1}{\max}\left({∆}_{{\alpha^{\prime }}_{ij}}\right)>\alpha_{thr}\;\\ \mathrm{non}-\mathrm{boundary}\;\mathrm{point},\;\mathrm{other} \end{array}\right.$$

where $\alpha_{thr}$ is the threshold value of the difference in adjacent azimuth angles

Let the point set of the target voxel be $P^{B}={\left\{P_{i}^{B}\right\}}_{i}^{k}$, where $P_{i}^{B}$ represents the i-th point in the target voxel, and i is the index of the point, with the value range from 1 to k.

Let the point sets of other voxels be $Q_{1}^{B}={\left\{Q_{1j}^{B}\right\}}_{j=1}^{n1},Q_{2}^{B}={\left\{Q_{2j}^{B}\right\}}_{j=1}^{n2}\ldots Q_{m}^{B}={\left\{Q_{mj}^{B}\right\}}_{j=1}^{nm}$, respectively. Here, $Q_{lj}^{B}$ represents the j-th point in the l-th voxel $l=\left(1,2,3\ldots m\right)$, j is the index of the point, and nl is the number of points in the l-th voxel.

Let the distance between the i-th point $P_{i}^{B}$ in the target voxel and the j-th point $Q_{lj}^{B}$ in the l-th voxel be defined as $d\left(P_{i}^{B},Q_{lj}^{B}\right)$:

(10) $$d\left(P_{i}^{B},Q_{lj}^{B}\right)=\sqrt{\sum_{s=1}^{d}{\left(p_{is}^{B}-q_{ljs}^{B}\right)}^{2}}$$

where $p_{is}^{B}$ and $q_{ljs}^{B}$ are the coordinates of points $P_{i}^{B}$ and $Q_{lj}^{B}$ in the s-th dimension, respectively, and d is the dimensionality of the space.

For the target supervoxel, we utilize the K-nearest neighbor algorithm to identify m candidate neighboring supervoxels. The distances from each point in the target supervoxel to all points in every other supervoxel are computed, obtaining m distance set ${\left\{D_{il}\right\}}_{l=1}^{m}$:

(11) $$D_{il}={\left\{d\left(P_{i}^{B},Q_{lj}^{B}\right)\right\}}_{j=1}^{nl}\;$$

For each point $P^{B}={\left\{P_{i}^{B}\right\}}_{i}^{k}$ in the target voxel, the minimum value in each distance set $D_{il}$ is found, as shown in Equation (12):

(12) $$\mathrm{Min}\left(D_{il}\right)=\min{\left\{d\left(P_{i}^{B},Q_{lj}^{B}\right)\right\}}_{j=1}^{nl}$$

Based on Equation (12), the adjacent supervoxels $S_{j_{i}^{*}}^{B}$ for each boundary point within the target supervoxel are identified, as shown in Equation (13)

(13) $$j_{i}^{*}=arg\;{min}_{j=1}^{m}\;\mathrm{Min}\left(D_{il}\right)$$

**Figure 2:**
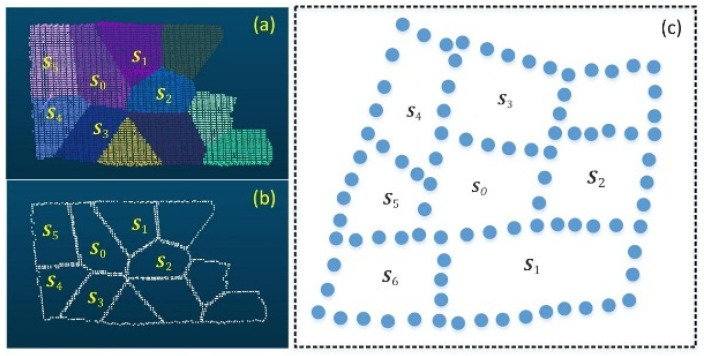
Neighboring voxel blocks of the target supervoxel: (**a**) different supervoxels, (**b**) different supervoxel boundaries, (**c**) schematic diagram of the target supervoxel boundary and the neighboring supervoxel boundary.

After deduplicating the voxel blocks $S_{j_{i}^{*}}^{B}$ corresponding to the minimum distances of all boundary points of the target supervoxel blocks, the final set of adjacent voxel blocks $S_{i}^{B}$ is obtained, as shown in Figure 2.

(14) $$S_{i}^{B}={⋃}_{i=1}^{k}\left(arg\;{min}_{j=1}^{m}\;\mathrm{Min}\left(D_{il}\right)\right)$$

### 3.2. Cluster Fusion of Supervoxels

In this paper, weight calculations are performed for all adjacency pairs of supervoxels by leveraging the normal information of supervoxels. Specifically, clustering of supervoxel adjacency pairs with smaller weights is prioritized to achieve plane segmentation. The supervoxels generated by the TBBS algorithm can better preserve object boundaries. Moreover, significant differences exist between adjacent supervoxel pairs of different planes, and these differences can be effectively distinguished using the normal information.

For each point $q_{j}$ in any supervoxel block, its mean $\bar{q}$ is computed, as shown in Equation (15):

(15) $$\bar{q}=\frac{1}{k}\sum_{j=1}^{k}q_{j}$$

Based on Equation (15), the covariance matrix $C_{1}$ is constructed, as shown in Equation (16):

(16) $$C_{1}=\frac{1}{k}\sum_{j=1}^{k}(q_{j}-\bar{q}){\left(q_{j}-\bar{q}\right)}^{T}$$

where $q_{ij}$ represents the coordinates of points in the neighborhood, and $\bar{q}$ is the average coordinates of these points. Eigenvalue decomposition is performed on the covariance matrix $C_{1}$ to obtain the eigenvalues $\lambda_{1}\leq \lambda_{2}\leq \lambda_{3}$ and their corresponding eigenvectors $\nu_{1},\nu_{2},\nu_{3}$. The eigenvector $\nu_{1}$ corresponding to the smallest eigenvalue $\lambda_{1}$ is the estimated normal vector of the supervoxel block point cloud. Using Equations (15) and (16), the normal vectors of adjacent supervoxels can be computed as $\vec{v_{i}}=\left(a_{x},a_{y},a_{z}\right)$ and $\vec{v_{j}}=\left(b_{x},b_{y},b_{z}\right)$. These normal vectors can be used to derive the first constraint condition $M_{ij}$, as shown in Equation (17):

(17) $$M_{ij}=\left|\vec{v_{i}}\times \vec{v_{j}}\right|=\sqrt{({a_{y}b_{z}-a_{z}b_{y})}^{2}+{\left(a_{z}b_{x}-a_{x}b_{z}\right)}^{2}+{\left(a_{x}b_{y}-a_{y}b_{x}\right)}^{2}}$$

Determine the eigenvector $\nu_{3}$ corresponding to the largest eigenvalue $\lambda_{3}$ based on Equation (16), and use this eigenvector as the projection plane vector for the space flipping of the supervoxel block point cloud. Using the projection vector $\nu_{3}$ and the center point $\bar{q}$ of the supervoxel block point cloud, the equation of the projection plane is determined:

(18) $$\tilde{a}x+\tilde{b}y+\tilde{c}z+d=0$$

where $\left[\tilde{a},\tilde{b},\tilde{c}\right]$ represents the projection vector $\nu_{3}$, and $d=-\nu_{3}{\bar{q}}^{T}$.

The point cloud of the supervoxel block is projected onto the plane, forming the projected point cloud ${p^{\prime }}_{s}=\left({x^{\prime }}_{s},{y^{\prime }}_{s},{z^{\prime }}_{s}\right)$. Subsequently, it is converted to the 2D space of the xoy plane, as shown in the following steps:

The cross product of the projection vector $\nu_{3}$ and the unit normal vector of the xoy plane $\nu_{z}=\left(0,0,1\right)$ is calculated:

(19) $$U=\nu_{z}\times \nu_{3}$$

The U is normalized, as described in Equation (20):

(20) $$E_{u}=\frac{U}{\left|U\right|}$$

The transformation angle is obtained based on the quaternion conversion method:

(21) $$\varphi =arccos\left(\frac{\nu_{3}\cdot \nu_{z}}{\left|\nu_{3}\right|\left|\nu_{z}\right|}\right)/2$$

Equations (20) and (21) are combined to obtain the quaternion (22):

(22) $$Q=\left[\begin{array}{cc} cos\left(\varphi \right) & sin\left(\varphi \right) \end{array}*E_{u}\right]$$

Based on the quaternion from Equation (22), the transformation matrix for the 3D-to-2D conversion of the projected supervoxel point cloud is obtained, as shown in Equation (23):

(23) $$R=\left[\begin{array}{ccc} 2{q\left(1\right)}^{2}-1+2{q\left(2\right)}^{2} & 2\left(q\left(2\right)q\left(3\right)+q\left(1\right)q\left(4\right)\right) & 2\left(q\left(2\right)q\left(4\right)-q\left(1\right)q\left(3\right)\right)\\ 2\left(q\left(2\right)q\left(3\right)-q\left(1\right)q\left(4\right)\right) & 2{q\left(1\right)}^{2}-1+2{q\left(3\right)}^{2} & 2\left(q\left(3\right)q\left(4\right)+q\left(1\right)q\left(2\right)\right)\\ 2\left(q\left(2\right)q\left(4\right)+q\left(1\right)q\left(3\right)\right) & 2\left(q\left(3\right)q\left(4\right)-q\left(1\right)q\left(2\right)\right) & 2{q\left(1\right)}^{2}-1+2{q\left(4\right)}^{2} \end{array}\right]$$

Based on Equation (23), the projected supervoxel block point cloud after the 3D-to-2D transformation can be obtained, as shown in Equation (24):

(24) $${P^{\prime }}_{R}={p^{\prime }}_{s}*R$$

**Figure 3:**
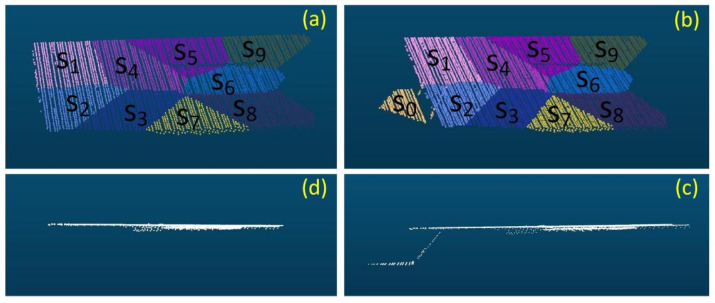
Geometric morphology of the target and neighboring supervoxels after projection space transformation. (**a**,**b**) point cloud of the target and neighboring supervoxel blocks, (**c**) geometric morphology of the supervoxel block point cloud after projection space transformation for a coplanar pair, showing a coherent linear structure, (**d**) geometric morphology for a non-coplanar but adjacent pair, showing two distinct linear clusters.

Based on Equation (24), the geometric feature distribution of the target and neighboring supervoxel 2D points in the 2D state can be obtained, as shown in Figure 3.

Based on the projected point cloud data obtained from Equation (24), the centroid of the projected point cloud $c={\left(c_{x},c_{y},c_{z}\right)}^{T}$ is computed, as shown in Equation (25).

(25) $$\left\{\begin{array}{c} c_{x}=\frac{1}{n}\sum_{i=1}^{n}x_{i}\\ c_{y}=\frac{1}{n}\sum_{i=1}^{n}y_{i}\\ c_{z}=\frac{1}{n}\sum_{i=1}^{n}z_{i} \end{array}\right.$$

Using the centroid point cloud, the projection point cloud is centralized as shown in Equation (26):

(26) $${P^{\prime }}_{Rc}={p^{\prime }}_{Ri}-c$$

The covariance $C_{2}$ elements $C_{jk}\;\left(j,k=x,y,z\right)$ for the points ${p^{\prime }}_{Rc}$ after centroid translation are calculated as follows:

(27) $$C_{2}=\left[\begin{array}{ccc} C_{xx} & C_{xy} & C_{xz}\\ C_{yx} & C_{yy} & C_{yz}\\ C_{zx} & C_{zy} & C_{zz} \end{array}\right]\;$$

where:

(28) $$\left\{\begin{array}{c} C_{xx}=\sum_{i=1}^{k}{\left(x_{i}-c_{x}\right)}^{2}\\ \;C_{xy}=C_{yx}=\sum_{i=1}^{k}\left(x_{i}-c_{x}\right)\left(y_{i}-c_{y}\right)\;\\ C_{xz}=C_{zx}=\;\sum_{i=1}^{k}\left(x_{i}-c_{x}\right)\left(z_{i}-c_{z}\right)\\ C_{yy}=\sum_{i=1}^{k}{\left(y_{i}-c_{y}\right)}^{2}\\ C_{yz}=C_{zy}=\sum_{i=1}^{k}\left(y_{i}-c_{y}\right)\left(z_{i}-c_{z}\right)\\ C_{zz}=\sum_{i=1}^{k}{\left(z_{i}-c_{z}\right)}^{2} \end{array}\right.$$

Eigenvalue decomposition is performed on the covariance matrix $C_{2}$, as shown in Equation (29):

(29) $$C_{2}={U\Sigma U}^{T}$$

where $U\in R^{3\times 3}$ is the eigenvector matrix, and its column vectors are the eigenvector; Σ is the real diagonal matrix, $\Sigma \in R^{3\times 3}$, with the eigenvalue on the diagonal, arranged in descending order; Based on the results of the eigenvalue decomposition, the third column of the eigenvector matrix U is taken as the direction vector d of the fitted line, i.e.,:

(30) $$d=\;U\left(:,3\right)$$

Let the starting point of the fitted line be o (i.e., the centroid c), and the direction vector be d. Let a point in the point cloud be p. The first step is to calculate the vector from point p to the starting point of the line:

(31) v=p−o

The projection length t of point p onto the line is calculated by Equation (32):

(32) $$t=\frac{v^{T}d}{{\left\|d\right\|}^{2}}\;$$

The projection vector $p_{proj}$ of point p onto the line is calculated by Equation (33):

(33) $$p_{proj}=td$$

The error of point p with respect to the fitted line is computed according to Equation (34):

(34) $$e=\left\|v-p_{proj}\right\|$$

The overall fitting error for an adjacent supervoxel block with m points is:

(35) $$\bar{e}=\frac{1}{m}\sum_{i=1}^{m}e_{i}$$

The rationale for employing this projection-line fitting constraint is rooted in its ability to capture global geometric consistency, which is a key advantage over purely local measures. Standard normal-based constraints [17,23] evaluate the angle between the average normals of two supervoxels. In stepwise non-coplanar scenarios, such as the adjacent steps of a staircase, the surfaces are nearly parallel. Consequently, the angle between their normals approaches zero, causing a normal-based constraint to erroneously suggest that they belong to the same plane, leading to under-segmentation. Curvature-based measures [27] are similarly challenged, as they are sensitive to local noise at the sharp step edge and do not provide a reliable signal for separation. The proposed projection-line fitting method overcomes this by evaluating the spatial distribution of the entire point set of adjacent supervoxels after transformation. As illustrated in Figure 3, when two supervoxels are truly coplanar (Figure 3c), their points, when projected onto the target’s principal plane, will be intermingled and well-represented by a single line, resulting in a low mean fitting error $\bar{e}$. Conversely, for non-coplanar but adjacent supervoxels (Figure 3d), their projections form two spatially distinct clusters in the 2D space. Attempting to fit a single line to this combined projection yields a high fitting error $\bar{e}$, which serves as a robust indicator that they belong to different planar regions. This error metric acts as a global constraint, reflecting the overall geometric harmony between two supervoxels and is inherently more resilient to local boundary noise and the geometric ambiguities that plague purely local methods in complex configurations like stepwise and intersecting planes.

Based on the above two constraints $M_{ij}$ and $\bar{e}$ for supervoxel fusion, they can be summarized as Equation (36):

(36) $$CC\in \left\{M_{ij}\cap \bar{e}|M_{ij}<0.5,\bar{e}<\frac{r}{10}\right\}$$

where r is the resolution of the supervoxel.

The merging criteria defined in Equation (36) determine when two adjacent supervoxels are geometrically consistent enough to belong to the same planar surface. However, a complete segmentation method requires a well-defined procedure for applying these criteria iteratively to grow planar regions from initial seeds, handle chains of supervoxels, and resolve competing merge candidates. The complete merging algorithm consists of the following steps:

(1) Initialization: All supervoxels are marked as unvisited.
(2) Seed Selection: An unvisited supervoxel is selected as the initial seed for a new plane cluster.
(3) Region Growing: Starting from the seed, the algorithm iteratively merges adjacent supervoxels that satisfy the merging condition (Equation (36)).
(4) Cluster Formation: When no further merges are possible from the current cluster, the cluster is finalized, and all its constituent supervoxels are marked as visited.
(5) Termination: Steps (2)–(4) are repeated until all supervoxels have been visited.

For all adjacent voxel pairs, the set $G^{m}=\{A^{m},W^{m}\}$, is defined, where $A^{m}$ consists of the two supervoxels in the adjacent voxel pair, and $W^{m}=\{w_{i,j}^{m}\}$ represents their corresponding weights. For instance, supervoxels $S_{1},S_{2}\in A^{m},w_{1,2}^{m}=1$. The weights $W^{m}$ are used to quantify the normal vector dissimilarity between the supervoxels in region $A^{m}$. At the beginning of the iterative process, the algorithm first clusters the pair $A^{m}$ associated with $min\{W^{m}\}$, generating a new supervoxel block $S_{new}$. This $S_{new}$ inherits all adjacency relationships from the original supervoxel blocks in $A^{m}$, and new weights are computed for these relationships, resulting in an updated set $G^{m-1}=\{A^{m-1},W^{m-1}\}$. After k iterations, if $min\left\{W^{m-k}\right\}>t$ (where t is a predefined weight threshold) in the adjacency set $G^{m-K}=\left\{A^{m-k},W^{m-k}\right\}$, the iteration terminates.

## 4. Experiment Analysis

This section evaluates the performance of the proposed method on multiple real-world 3D point cloud datasets acquired from LiDAR and RGB-D sensors. Detailed implementation specifications are provided, covering parameter settings, evaluation metrics, and dataset characteristics. The experiment is mainly implemented using the PCL library in C++, and the experimental equipment is configured with an i5-9300F CPU processor.

### 4.1. Evaluation Metrics

To quantitatively and qualitatively assess the performance of the proposed method, we adopt widely used metrics including Precision, Recall, and F1-score, which measure the overlap between segmented planes and ground truth annotations.

However, standard metrics may not fully capture geometric fidelity in complex scenarios involving stepwise transitions or intersecting planes—common challenges in real-world sensor data. To address this, we further employ a plane-aware segmentation evaluation metric (formally defined in Equations (37) and (38)) that jointly penalizes over-segmentation (splitting a single true plane into multiple segments) and under-segmentation (merging distinct planes into one). This metric provides a more comprehensive assessment of structural consistency in 3D plane segmentation tasks.

(37) $$Index=\frac{1}{k}\sum_{i=1}^{k}P_{i}$$

(38) $$P_{i}=\frac{T_{i}}{T_{i}+F_{i}^{R}+F_{i}^{S}}$$

where $T_{i}$ is the number of points in the segmented point cloud that perfectly correspond to the matched real block point cloud, $F_{i}^{R}$ is the number of points in the real block point cloud that cannot correspond to the matched segmented point cloud, and $F_{i}^{S}$ is the number of points in the segmented point cloud that cannot correspond to the matched real block point cloud:

For the real point cloud set $\Phi^{T}=\left\{S_{1},S_{2},\ldots ,S_{m}\right\}$ and the segmented block point cloud set $\Phi =\left\{S_{1},S_{2},\ldots ,S_{l}\right\}$, iterate through each segmented block point cloud $S_{i},i=1,2,\ldots ,l$ and check the correspondence with all real block point clouds in $\Phi^{T}=\left\{S_{1},S_{2},\ldots ,S_{m}\right\}$. If there is a corresponding point, i.e., the point-to-point distance is 0, then determine that the segmented block point cloud matches the real block point cloud.

Precision measures the proportion of true plane points among all the points classified as plane points, as shown in Equation (39):

(39) $$Precision=\;\frac{TP}{TP+FP}$$

In plane segmentation, Intersection over Union (IoU) is a commonly used metric to measure the degree of overlap between the predicted region and the ground truth region. It is widely used in computer vision tasks such as object detection and segmentation. The formula is as follows:

(40) $$IoU=\frac{A\cap B}{A_{area}+B_{area}-\left(A\cap B\right)}$$

Recall (or recall rate) is used to measure the proportion of true plane points identified by the model out of all the actual plane points, as shown in Equation (41):

(41) $$Recall=\frac{TP}{TP+FN}$$

The F1-score is the harmonic mean of precision and recall. It is particularly useful in cases of imbalanced data, as the precision and recall abilities of the model are simultaneously considered, as shown in Equation (42):

(42) $$F1=2\times \frac{Precision\times Recall}{Precision+Recall}$$

where TP (True Positives) represents the number of correctly classified plane points, *FP* (False Positives) represents the number of non-plane points incorrectly classified as plane points, and *FN* (False Negatives) represents the number of plane points incorrectly classified as non-plane points.

### 4.2. Parameter Settings

In this paper, Table 1 presents the main parameter settings used during the experimental process. The parameter r controls the resolution of supervoxel generation, which plays a crucial role in the final segmentation results. The parameter k represents the search range for the k-nearest neighbors of each point, which is utilized in various aspects such as the normal calculation of point cloud data during the experiment. In this method, the adjacency relationships of supervoxels are obtained by calculating the distances between the current supervoxel and the boundary points of all other supervoxels. However, computing the distances to the boundary points of all supervoxels will entail a substantial computational burden. To address this issue, this paper employs the parameter $k_{c_{n}}$ to reduce the search range for each supervoxel, thereby decreasing the amount of computation. When setting $k_{c_{n}}$, it is typically configured as twice the value of r. The parameter t serves as the first constraint in this method for controlling the clustering of supervoxels. By leveraging the magnitude of the cross product of the normal vectors of supervoxel blocks, different planes can be distinguished. For non-planar structures, setting t to 0.5 achieves good segmentation results. The parameter e represents the second constraint in this method, further optimizing the clustering results of supervoxels and reducing the phenomenon of over-segmentation. During the experiment, it is usually set to one-tenth of r.

**Figure 4:**
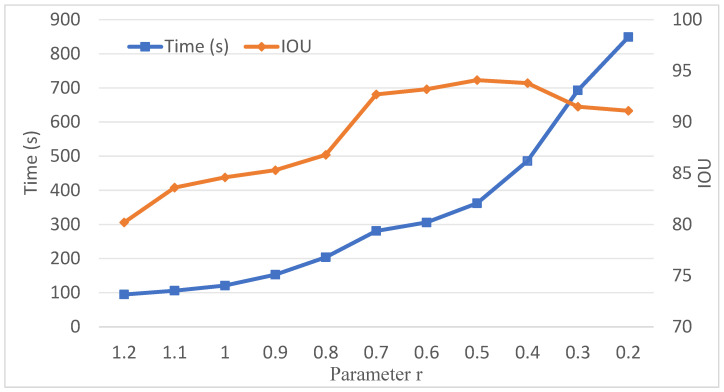
The influence trend of parameter r on running time and segmentation accuracy.

The parameter r listed in Table 1 serves as a critical tuning variable. Its magnitude directly governs the number of generated supervoxels, which in turn dictates the computational cost of the proposed method. Specifically, the supervoxel count influences not only the overall runtime but also the time required for boundary point extraction and adjacency determination. Consequently, variations in r significantly impact both computational efficiency and segmentation accuracy. To evaluate this trade-off, experiments were conducted on wall point clouds using various values of r, with the results illustrated in Figure 4.

As illustrated in Figure 4, the computational time exhibits an exponential increase as the parameter r decreases. However, the segmentation accuracy, measured by Intersection over Union (IoU), does not monotonically improve with smaller values of r. Instead, the IoU follows a trend of initial improvement followed by a slight decline. Specifically, the maximum IoU is achieved when r is reduced from 1.2 to 0.5. Further reduction of r beyond this range yields no significant gain in accuracy but incurs a substantial increase in computational cost.

### 4.3. Results

**Figure 5:**
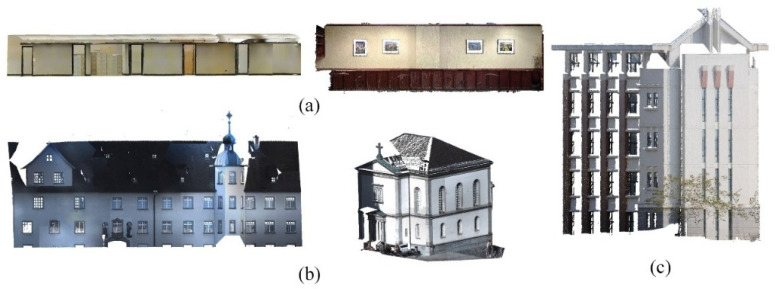
Experimental dataset. (**a**) S3DIS; (**b**) SEMANTIC3D.NET; (**c**) Haiyun hotel.

The experiments are conducted on three real-world 3D point cloud datasets acquired from commercial 3D sensors, along with controlled synthetic data for ablation studies. The first dataset is the Stanford Large-Scale 3D Indoor Spaces (S3DIS) (Armeni et al. [33]), captured using a Matterport RGB-D camera. It comprises six large-scale indoor areas with diverse layouts. In this work, we focus on corridor and office scenes (Figure 5a). The second dataset is Semantic3D.net (Hackel et al. [34]), one of the largest terrestrial laser scanning benchmarks, containing over 4 billion points across urban, rural, and forested environments. We select building facades from their urban scans for evaluation (Figure 5b). The third dataset consists of indoor scans of the Haiyun Hotel in Xiamen, collected by Lin [35] using a static LiDAR scanner (e.g., FARO Focus or equivalent). This dataset provides complex architectural structures with abundant planar surfaces (Figure 5c). Additionally, synthetic point clouds generated from CAD models are employed solely to analyze algorithm behavior under idealized conditions, while all primary results are reported on real sensor data.

**Figure 6:**
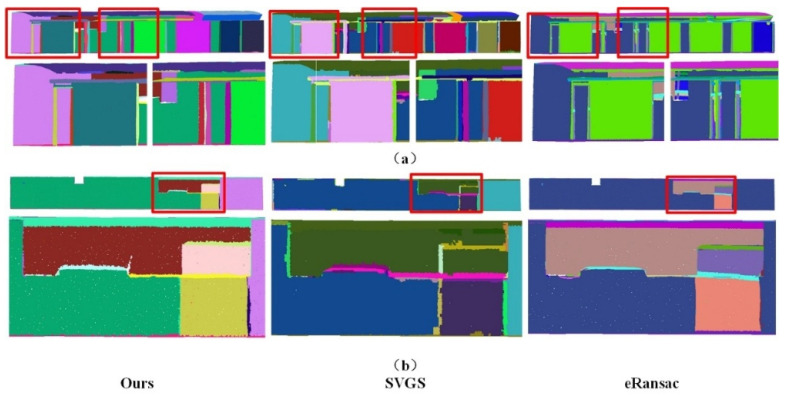
Data 1. Segmentation results of corridor data by different methods under different perspectives. (**a**) front view and details, (**b**) rear view and details.

**Figure 7:**
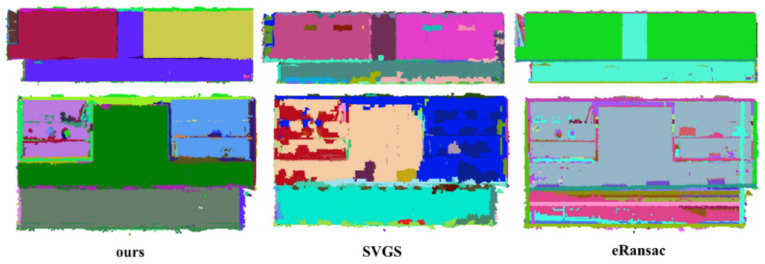
Data 2. Segmentation results of the office room by different methods.

**Figure 8:**
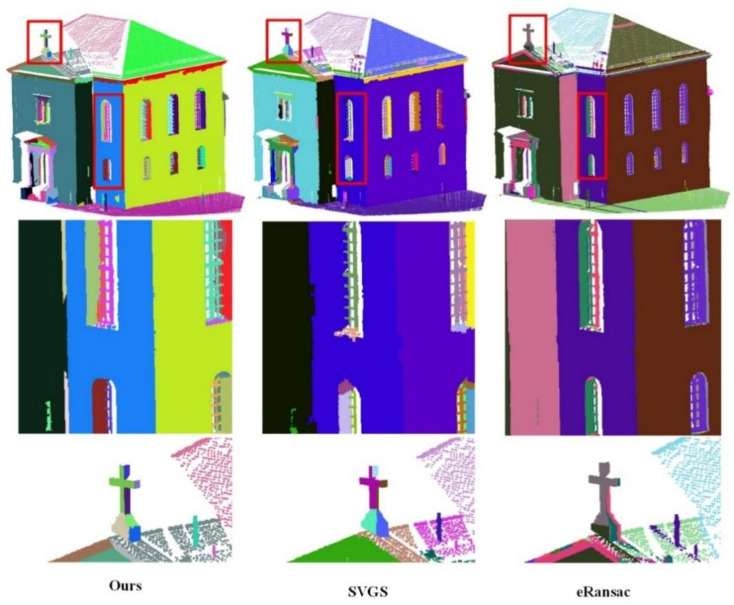
Data 3. Segmentation results of the church by different methods. The first row shows the overall segmentation results, and the second and third rows display the details.

**Figure 9:**
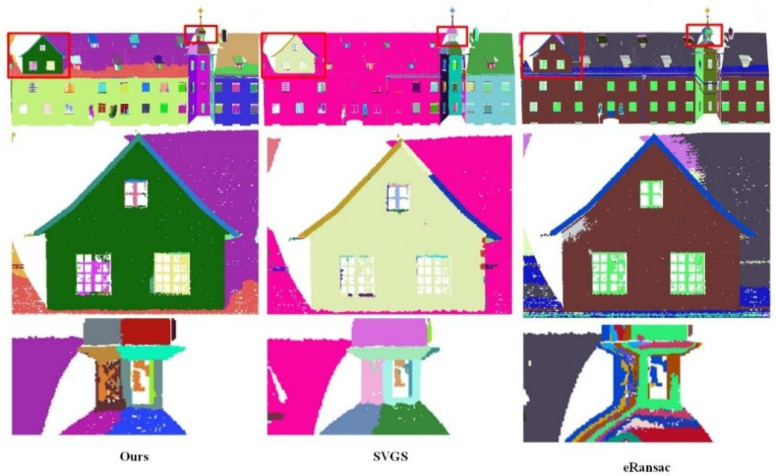
Data 4. Segmentation results of the Tower room by different methods. The first row shows the overall segmentation results, and the second and third rows display the details.

**Figure 10:**
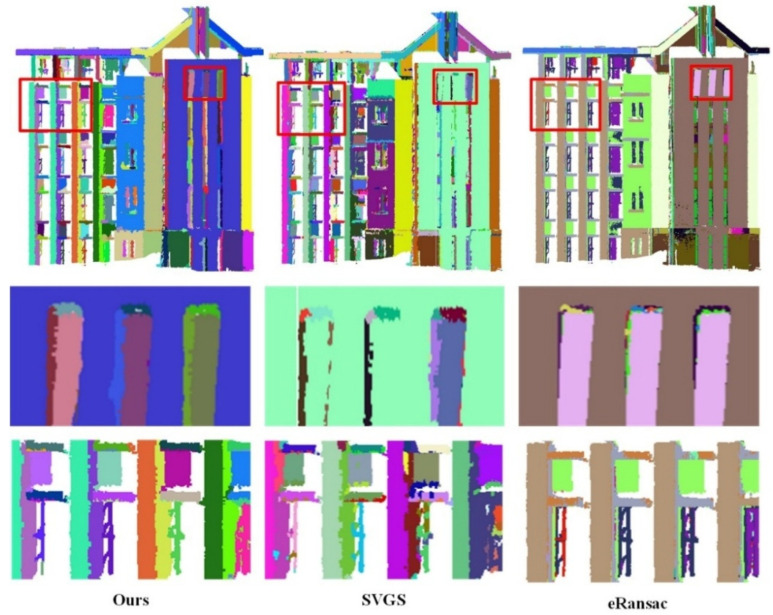
Data 5. Segmentation results of the Haiyun hotel by different methods. The first row shows the overall segmentation results, and the second and third rows display the details.

**Figure 11:**
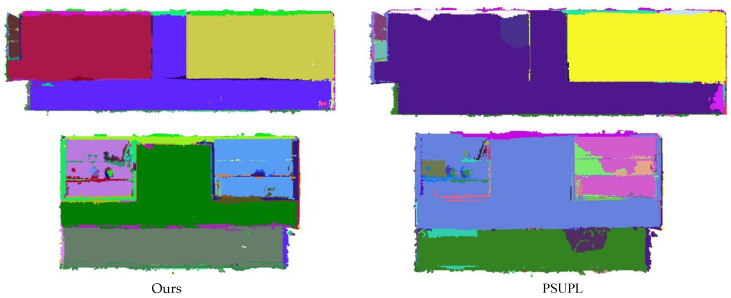
Comparison of the segmentation results between the PSUPL and proposed method.

**Figure 12:**
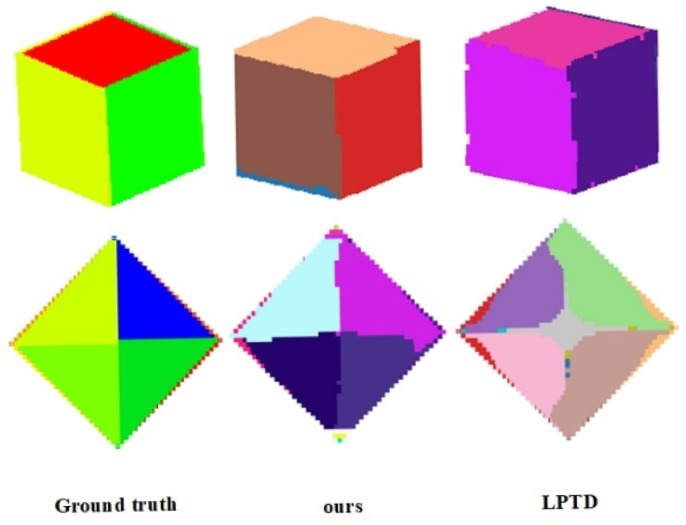
Data 6 and Data 7. Segmentation results of the simulated data of regular shape by different methods.

**Figure 13:**
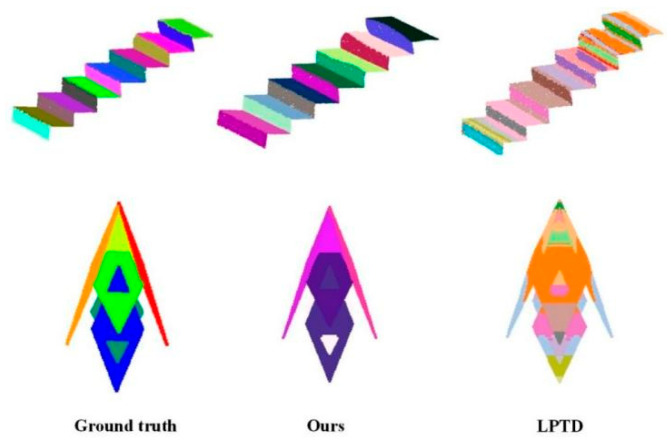
Data 8 and Data 9. Segmentation results of the simulated data of irregular shapes by different methods.

Qualitative Analysis: As shown in Figure 6, Figure 7, Figure 8, Figure 9, Figure 10, Figure 11, Figure 12 and Figure 13, all visualization results are in color, where the same color represents the same plane. Specifically, as illustrated in Figure 6 and Figure 7, within the S3DIS dataset, comparisons were made between our proposed method and the other two plane segmentation methods, namely SVGS [36] and eRansac [37], using different sides of the data. As shown in Figure 6, in contrast to the other two algorithms, the proposed method accurately segments different planes and preserves their integrity, particularly for step-shaped irregular surfaces. The SVGS algorithm tends to under-segment and is easily influenced by noise in the point cloud data. Consequently, some isolated points in the same plane appear in the segmentation results. Moreover, it fails to effectively segment step-shaped irregular surfaces. The eRansac algorithm cannot accurately segment different planes, is highly sensitive to noise, and fails to preserve the integrity of the planes.

As shown in Figure 8, Figure 9 and Figure 10, the visualized results are presented using the main view of the experimental data and a close-up view of the red bounding box. As shown in Figure 8, for data with fewer noise points, both the SVGS and eRansac algorithms can accurately segment most of the planes of the church. However, the proposed algorithm provides more precise segmentation in terms of details compared to the other two. As shown in the first red bounding box, the proposed algorithm can accurately segment all the planes within the window. The SVGS algorithm can segment the window frame but fails to accurately segment the wall inside the window. The eRansac algorithm can segment the planes but cannot accurately classify different planes. In its visualized results, all windows and the walls inside them are segmented into the same category. As shown in the second red bounding box, compared to SVGS and eRansac, the proposed algorithm provides more accurate segmentation of the cross, successfully segmenting each face of the cross and maintaining good boundary relationships between the faces.

In Figure 9, the SVGS algorithm fails to segment the main planes of the experimental data, resulting in severe under-segmentation. It also cannot accurately segment the stepped, non-parallel planes, such as the wall and window in the first red bounding box. The eRansac algorithm provides relatively accurate segmentation of the main planes, but it cannot classify them accurately. Moreover, because the algorithm depends on parameter settings, its segmentation of smaller planes is significantly inferior to the other two algorithms. For instance, at the tower location in the second red bounding box, it fails to effectively segment the angular planes with noise.

In Figure 10, despite the presence of a large amount of noise in the experimental data, the proposed method still demonstrates excellent robustness. It accurately segments the different planes of the building, showing a clear advantage in handling stepped non-parallel planes at various structural locations. The first bounding box contains a very complex plane structure, yet the proposed method still performs a detailed division, restoring the building’s original physical structure. In contrast, SVGS encounters many instances of under-segmentation or over-segmentation when dealing with noisy and complex structures. eRansac lacks the ability to handle such complex scenes and fails to distinguish the planes effectively. In the second red bounding box, at the protruding part of the wall, the proposed method not only segments the wall well but also effectively distinguishes the various side faces at the connection points. In SVGS, under-segmentation occurs for this type of non-parallel plane, failing to maintain clear plane boundaries. While eRansac can segment stepped non-parallel planes, it does not achieve good segmentation of the side faces at the protruding areas.

In addition, the method proposed in this paper is compared with the latest PSUPL [31] and LPTD [38] method with different point cloud, as shown in Figure 11, Figure 12 and Figure 13.

As clearly shown in Figure 11, for the first wall, the proposed method achieves more accurate segmentation by distinguishing between different wall layers, whereas the PSUPL method merges adjacent walls, resulting in under-segmentation. Similarly, for the second wall, the proposed method successfully segments the central wall section, while the PSUPL method erroneously fuses the central wall with the neighboring walls.

Figure 12 and Figure 13 present a controlled evaluation on synthetic point clouds generated from CAD models with known ground-truth plane annotations. This setup allows us to isolate algorithmic behavior under idealized conditions, free from sensor noise or occlusion. We compare our method with LPTD [38], which computes pairwise tangent plane distances to construct an n×n LPTD matrix, applies Multidimensional Scaling (MDS) for dimensionality reduction, and finally performs adaptive DBSCAN clustering for plane segmentation. While LPTD offers an elegant geometric formulation, its reliance on a full pairwise distance matrix leads to high memory consumption and quadratic computational complexity, limiting its scalability to large-scale or real-time sensing applications. Moreover, as shown in Figure 12 and Figure 13, LPTD struggles with complex geometric configurations—particularly stepwise non-coplanar surfaces and intersecting planes—exhibiting significant over-segmentation (splitting single planes) and under-segmentation (merging distinct planes). In contrast, the proposed method maintains structural integrity across these challenging cases, demonstrating superior robustness and practicality for deployment in real-world 3D sensing systems where computational efficiency and geometric fidelity are critical.

Quantitative Analysis: In order to evaluate the segmentation performance of the proposed algorithm, manual labeling of the experimental data was conducted. The results are compared and analyzed using five metrics: accuracy, recall, precision, F1 score, and the specific quantitative data. Table 2 shows the corresponding information of the input point cloud data as well as the specific quantitative statistics. Here, we start with the first set of corridor data in this chapter and proceed with sequential numbering.

As shown in Table 2, the data indicates that this method outperforms other segmentation algorithms. Specifically, for point cloud data with low noise, such as data1, the algorithm demonstrates excellent plane segmentation capability, greatly preserving the integrity of each plane in the original point cloud data’s physical structure. For data with more noise, such as data 2 and data 5, the algorithm also exhibits outstanding robustness. In contrast, the SVGS algorithm shows poor robustness against noise and clutter, with the main reason being that the supervoxel connectivity does not accurately reflect the original physical structure of the point cloud data. The supervoxel connections are severely affected by noise, which prevents the preservation of plane integrity during reclustering. The eRansac algorithm lacks the ability to segment parallel planes, which results in it grouping many parallel but non-parallel planes together, leading to very low accuracy during segmentation.

Quantitative statistics on the segmentation results for both regular and irregular simulated point cloud scenarios are presented in Table 3. Given that the simulated data exhibits distinct block-level matching features, the evaluation focuses on four core metrics: Intersection over Union (IoU), Recall, Precision, and F1-score.

For the Regular Simulated Scenarios (Data 6, Data 7): Table 3 shows that both the proposed method and the LPTD method achieve high performance metrics. Although LPTD yields a marginally higher IoU in Data 6, it underperforms the proposed method in Recall, Precision, and F1-score. This indicates that while both methods are effective for simple, regular geometric structures, the proposed method demonstrates superior comprehensive performance. For the Irregular Simulated Scenarios (Data 8, Data 9): The proposed method maintains robust performance with all metrics exceeding 95%. In stark contrast, the LPTD method suffers a drastic decline in performance. Specifically, in the complex scenario involving asymmetric intersections of multiple planes (Data 9), LPTD’s IoU and F1-score plummet to 50.7% and 48.5%, respectively. This significant degradation suggests that LPTD lacks the capacity to effectively characterize complex geometric relationships, rendering it inadequate for handling irregular structures such as stepped skew surfaces and multi-plane intersections.

It should be noted that the synthetic data shown in Figure 12 and Figure 13, while useful for controlled evaluation of geometric accuracy under ideal conditions, do not fully represent the noise characteristics of real sensor data. The primary conclusions of this paper regarding method performance are drawn from the real sensor datasets (S3DIS, Semantic3D, and Haiyun Hotel), where the method demonstrates robust performance under realistic noise and sampling conditions.

### 4.4. Limitation Analysis

**Figure 14:**
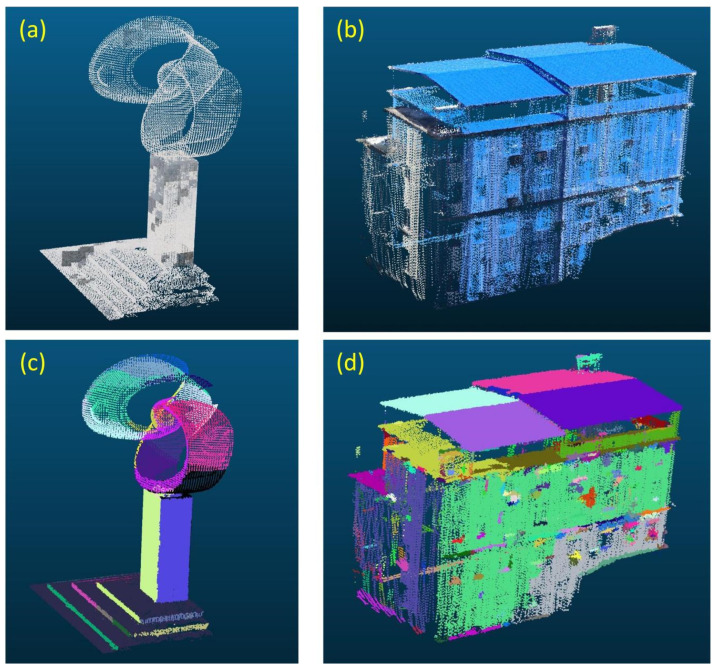
Segmentation results of surfaces and curved surface point clouds by the proposed method, (**a**,**b**) show the original point clouds of curved structures and sparse scenes, respectively; (**c**,**d**) present the corresponding segmentation results.

Since our approach is fundamentally based on supervoxel clustering for planar segmentation, its applicability to curved surfaces and sparse point clouds is potentially limited. To investigate these limitations, we applied the method to point cloud data of structures with curved geometries and to sparse building facade point clouds acquired by unmanned aerial vehicle (UAV). The resulting segmentation outcomes are illustrated in Figure 14.

As shown in Figure 14a, the structure exhibits a hybrid geometry: the upper section is distinctly curved, while the lower section consists of rectangular planar surfaces. The segmentation results in Figure 14c demonstrate that the proposed method successfully segments the planar point clouds in the lower section. However, for the curved structure in the upper section, the method fails to achieve a coherent segmentation, instead fragmenting the surface into multiple discrete patches. This limitation arises because the proposed approach is fundamentally designed for planar segmentation; consequently, it approximates curved surfaces as combinations of small planar facets rather than recognizing them as continuous curvatures.

## 5. Conclusions

This paper presents a robust plane segmentation method tailored for real-world 3D point clouds acquired from LiDAR and RGB-D sensors. By leveraging supervoxel generation and a boundary-aware adjacency model, the method effectively preserves geometric boundaries that are often degraded by sensor noise and sparse sampling. The subsequent clustering refinement—based on projection-line fitting errors—further enhances segmentation accuracy and structural integrity, particularly for stepwise non-coplanar surfaces and intersecting planes, which are common in indoor and urban environments. Experimental results on multiple real sensor datasets (including S3DIS, Semantic3D, and custom LiDAR scans) demonstrate that the proposed approach consistently outperforms state-of-the-art methods such as SVGS, eRANSAC, and LPTD in terms of precision, recall, and geometric fidelity. Notably, it achieves superior discrimination of adjacent parallel planes—a critical capability for applications of 3D scene understanding. Future work will focus on integrating this segmentation framework into real-time perception pipelines for resource-constrained embedded sensor platforms.

The proposed method is specifically designed for plane segmentation in man-made environments where planar surfaces dominate, such as indoor scenes, building facades, and urban structures. The projection-line fitting assumption underlying our approach relies on the planarity of surfaces and may not hold for significantly curved geometry. In such cases, the method would produce piecewise planar approximations rather than continuous curved surface reconstructions. Additionally, near-degenerate configurations—such as extremely thin structures or very closely spaced parallel planes—may fall below the resolution limit defined by sensor noise and supervoxel scale. These limitations are inherent to plane segmentation approaches and define the scope of applicability: our method is intended for scenes where planar structures are prevalent, which aligns with its target applications in 3D scene understanding.

## Figures and Tables

**Table 1:** Parameters of 3D plane segmentation.

Parameter	Value	Representation
r	~	Supervoxel resolution
k	15	K-Nearest Neighbor Search Range for Each Point
a	110°	The angle value for determining boundary points
$k_{c_{n}}$	2	The search range of candidate supervoxels
t	0.5	The control threshold for supervoxel clustering
e	0.1	The error control threshold for line fitting

**Table 2:** Performance Comparison of 3D Plane Segmentation Results.

	Methods	Index	$IoU\;\left(\%\right)$	$Recall\;\left(\%\right)$	$Precision\;\left(\%\right)$	$F1\;\left(\%\right)$
Data 1	ours	0.98	98.1	98.9	97.7	98.2
	SVGS	0.91	92.3	94.4	90.0	92.1
	eRansac	0.66	66.2	67.7	65.1	66.2
Data 2	ours	0.95	94.1	95.7	94.4	95.0
	SVGS	0.71	71.5	72.3	70.4	71.3
	eRansac	0.62	61.8	65.7	60.4	62.8
Data 3	ours	0.92	90.1	93.7	91.2	92.4
	SVGS	0.87	88.6	89.7	85.4	87.6
	eRansac	0.73	73.4	74.6	71.5	73.0
Data 4	ours	0.83	83.5	84.8	80.4	82.5
	SVGS	0.48	50.7	51.4	45.9	48.5
	eRansac	0.57	56.9	58.1	55.7	56.9
Data 5	ours	0.95	95.5	96.0	92.3	94.1
	SVGS	0.86	86.1	88.4	84.6	86.5
	eRansac	0.37	35.8	35.7	38.5	37.1

**Table 3:** Performance Comparison of 3D Plane Segmentation by different method.

	Methods	Index	$IoU\;\left(\%\right)$	$Recall\;\left(\%\right)$	$Precision\;\left(\%\right)$	$F1\;\left(\%\right)$
Data 6	ours	0.98	98.1	98.9	98.7	98.8
	LPTD	0.97	98.37	98.4	98.0	98.1
Data 7	ours	0.96	96.1	96.7	96.4	96.3
	LPTD	0.94	94.5	94.1	94.4	94.2
Data 8	ours	0.99	98.9	99.2	98.9	99.1
	LPTD	0.87	90.2	89.7	85.4	87.6
Data 9	ours	0.97	97.8	97.6	97.1	97.3
	LPTD	0.48	50.7	51.4	45.9	48.5

## Data Availability

The data used to support the findings of this study are available from the corresponding author upon request.
